# AC Electrothermal Effect in Microfluidics: A Review

**DOI:** 10.3390/mi10110762

**Published:** 2019-11-11

**Authors:** Alinaghi Salari, Maryam Navi, Thomas Lijnse, Colin Dalton

**Affiliations:** 1 Biomedical Engineering Graduate Program, Ryerson University, Toronto, ON M5B 2K3, Canada; maryam.navi@ryerson.ca; 2 Institute for Biomedical Engineering, Science and Technology (iBEST), St. Michael’s Hospital, Toronto, ON M5B 1T8, Canada; 3 Keenan Research Centre, St. Michael’s Hospital, Toronto, ON M5B 1T8, Canada; 4 Biomedical Engineering Graduate Program, University of Calgary, Calgary, AB T2N 1N4, Canada; thomas.lijnse@ucalgary.ca; 5 Electrical and Computer Engineering Department, University of Calgary, Calgary, AB T2N 1N4, Canada

**Keywords:** electrothermal, microelectrode, microfluidics, micromixing, micropump

## Abstract

The electrothermal effect has been investigated extensively in microfluidics since the 1990s and has been suggested as a promising technique for fluid manipulations in lab-on-a-chip devices. The purpose of this article is to provide a timely overview of the previous works conducted in the AC electrothermal field to provide a comprehensive reference for researchers new to this field. First, electrokinetic phenomena are briefly introduced to show where the electrothermal effect stands, comparatively, versus other mechanisms. Then, recent advances in the electrothermal field are reviewed from different aspects and categorized to provide a better insight into the current state of the literature. Results and achievements of different studies are compared, and recommendations are made to help researchers weigh their options and decide on proper configuration and parameters.

## 1. Introduction

Microfluidics is the precise control, and manipulation of fluids that are geometrically constrained to small, typically sub-millimeter, manufactured systems. Over recent decades, microfluidics has gained a great deal of attention in multiple fields, including medicine, chemistry, and biomedical engineering, due to its ability to perform multiplexing, automation, and high-throughput screening tasks. [1,2]. Due to the high surface-to-volume ratio of the fluid, and thus, the dominance of surface forces over inertial forces (i.e., low Reynolds number), fluid flow generation is a major challenge in microfluidic devices, as conventional pressure driven methods have poor efficiency in such devices [3,4]. The mechanisms of micro scale manipulation of fluids and particles can be categorized into two groups: mechanical, such as diaphragm-based devices, and non-mechanical, such as electrokinetic-based techniques. The presence of moving parts increases the risk of mechanical failure and can be incompatible with particulate flows, and thus, can limit the application of mechanical pumps for lab-on-a-chip devices [1,4,5]. Non-mechanical strategies, however, do not have these limitations. They can be integrated with microfluidic devices and also be used with particulate fluids [4,6,7]. Examples of non-mechanical methods include ultrasonic, direct current (DC) charge injection, and travelling wave driven electrohydrodynamic (EHD) micropumps [7,8,9,10].

Electrokinetics is a popular non-mechanical technique used for microfluidic fluid manipulation applications owing to its simple design and electronic automation [3]. Electrokinetic phenomena result from the interaction of an external electric field and induced electric charges. DC electrokinetics (DCEK), which has been studied over decades, requires relatively high voltages (i.e., on the order of several kilovolts) to operate which can limit its application in lab-on-a-chip devices [4,11,12]. alternating current (AC) electrokinetics (ACEK), however, which operates in low voltages (i.e., 1–20 V_rms_), has led to the development of devices being portable and capable of handling biofluids without engaging in unwanted chemical reactions [13,14]. Furthermore, with non-uniform fluid flow streamlines generated by ACEK, this mechanism can be used to mix fluids [6].

AC electrokinetics mainly includes the dielectrophoresis (DEP), AC electroosmosis (ACEO), and AC electrothermal effects (ACET) [15], each of which is explained briefly in the following sections. There have been many substantive review articles on micropumps [16,17], electrohydrodynamics [18], electrokinetics [3,19,20], and their subcategories [2,21], but a comprehensive review focused on the electrothermal effect in microfluidics is still missing in the literature. This review intends to study the advances in the utilization of the electrothermal effect in microfluidics from different aspects, namely: the electric field, temperature field, and velocity field. In addition, channel properties, conditions of numerical simulations, experimental setup, and applications of ACET effect are presented. Finally, we will mention the ongoing research directions and future potential opportunities for the electrothermal effect. The majority of the publications cited throughout the manuscript that have made major contributions are also summarized in Table A1. It should be noted that the study of the electrothermal effect in other mechanisms such as ACEO is not in the scope of this paper and can be found elsewhere [5,22]. Furthermore, strategies which are based on DC electric fields (e.g., DC electrophoresis) or non-electrical (e.g., magnetic) forces are not discussed here.

## 2. AC Electrokinetics

### 2.1. Dielectrophoresis

Dielectrophoresis arises from the interaction between a dipole moment on a particle and a non-uniform electric field [23]. If the particle has a polarizability higher than the surrounding medium, the DEP force exerting on the particle will be towards regions with a high electric field (positive DEP). For particles with lower polarizability, however, this force will be towards regions with a low electric field (negative DEP). This is demonstrated by the Clausius–Mossotti factor, which specifies the direction of the DEP force with respect to the electric field [24]. In addition to the permittivities of the particle and the medium, the magnitude of DEP force is also a function of the particle size. DEP force is directly related to the third power of the particle radius, and thus, is an ideal tool for separating, concentrating, and sorting particles, cells, and viruses [25,26,27,28,29,30,31]. Moreover, since DEP force scales with the gradient of the electric field, it decreases with the distance from electrodes, and the generated velocity is inversely proportional to the third power of distance [13]. Therefore, DEP is not an effective technique for handling particles of relatively small size (e.g., ≤1 µm) far from a strong electric field (e.g., a few micrometers away) [32]. Two excellent reviews on dielectrophoresis are [33,34].

### 2.2. AC Electroosmosis

AC electroosmosis is dependent on the formation of an electric double layer (EDL) at the interface of a liquid and solid substrate [35,36,37]. At an interface of a solid object and electrolyte fluid, due to the adsorption of ions onto the object surface it acquires charges, and as a result, an EDL forms inside the fluid near the surface [3,38]. When an electric field is applied to this system, the charges in this layer experience an electrostatic force, which can cause fluid motion. The rest of the fluid is then dragged into motion due to viscous forces.

Since the EDL thickness is inversely related to the fluid electrical conductivity, at relatively high conductivities (e.g., 84 mS·m^−1^), the EDL thickness becomes very small (e.g., <1 nm), which makes ACEO ineffective for the manipulations of biological fluids (1–2 S·m^−1^) [1,13,15,39,40,41]. In addition, ACEO is frequency-dependent, and increasing the actuation frequency beyond 100 kHz causes the ACEO effect to become invisible, since at high frequencies, the electric double layer is unable to form, and no fluid flow is generated [35]. Similarly, at very low frequencies, the double layer can completely screen the electric field, and thus, no net flow can be generated. ACEO has been developed and used in many forms to pump fluids or manipulate particles, namely biased ACEO for particle assembly and micropumping [32,42], micropumping of fluids [43,44], Travelling wave ACEO [45] and asymmetric ACEO micropumping [40], and DEP electrohydrodynamic particle trapping (i.e., ACEO in conjunction with DEP) [46,47]. As ACEO is only effective in relatively low frequencies, it is more prone to bubble generation and electrode deterioration resulting from electrochemical reactions, which can affect the electric field distribution and eventually damage the device [39]. Despite these limitations, there are many application-driven papers in the literature using ACEO [48,49,50,51], where modifications have been suggested to enhance ACEO applicability in fluids with electric conductivities up to 0.1 S·m^−1^ under the actuation of high frequencies and voltages. These modifications include the utilization of polarizable walls in induced-charge electroosmosis [52], AC faradic polarization [50], and nonlinear electroosmosis on curved surfaces [53], to name a few. An advantage of ACET is that it can be used for higher conductivities, i.e., over 1 S·m^−1^. More details of different strategies for implementation of ACEO in microfluidics can be found in [54,55]. DEP and ACET effects can be combined [13,56,57,58,59] to improve particle manipulations, as DEP has difficulty in manipulating submicron particles, and also is weak in areas far from electrodes where ACET is strong [13,56].

### 2.3. AC Electrothermal

Unlike ACEO and DEP, ACET has been shown to be very effective in biomedical applications which involve high conductivity biofluids, such as blood, urine, and saliva [60]. This is due to the fact that the ACET effect originates from a temperature gradient in the bulk of the fluid and not the fluid-electrode interface (i.e., the EDL). Fluids with higher conductivities can generate stronger microflows, and therefore, it is the most efficient electrokinetic mechanism for manipulating biological fluids with conductivities above 0.7 S·m^−1^ [1,4,13,14,39,60,61,62,63].

Emerging in 1960s [64,65], ACET has been widely used for fluid manipulations over the years [7,66], and is also referred to as induction EHD [67,68]. Despite similar flow patterns, the physics behind ACET and ACEO are different. ACEO is the result of the interaction of an electric double layer at the interface of the fluid-electrode and a non-uniform AC electric field, while ACET arises from the interaction of a temperature gradient in the bulk of the fluid and a non-uniform AC electric field. The source of the temperature gradient may be internal (i.e., Joule heating) or external (e.g., strong illumination, microheaters, etc.). Temperature gradients in the fluid lead to gradients in the electrical properties of the fluid, i.e., conductivity and permittivity, which induce free charge density. An electric force arising from the non-uniform electric field causes the free charges to move. As a result of shear stress, the surrounding fluid is also dragged into motion which produces microflows. Unlike ACEO, the ACET effect shows plateaus in force in a wide frequency range (10–10^11^ Hz) [39,69]. With ACET, the fluid velocity is steadier and more predictable at different frequencies compared to ACEO and DEP. In general, ACET flow can be generated in frequencies above 100 kHz and salt concentrations of above 10^−2^ mol·dm^−3^, whereas ACEO is more common at low frequencies and salt concentrations of 10^−2^ mol·dm^−3^ and below [62]. Despite the fact that fluid heating is crucial for the ACET effect, ambient heat conduction helps dissipating energy so that the temperature rise in the bulk of the fluid is typically low (ΔT < 5 K), which is safe for biofluids [11,13,14,41]. The ACET force is proportional to the temperature gradient |∇T| and not the temperature rise [1].

In order to generate AC electric fields required for inducing the electrothermal effect, microfabricated electrode arrays are commonly used. Employing a symmetric pair of electrodes at the bottom of a microfluidic channel can induce two symmetric sets of microvortices above the electrodes, and thus, no net flow can be generated [70]. For pumping applications, however, the electrode symmetry needs to be broken. Since the electrothermal force is a function of the electric field and temperature gradient, asymmetry may be achieved by manipulating either or both of these factors. This will be discussed in more details in the following sections. Typically, due to its simple implementation, imposing geometry asymmetry to microelectrodes is the most common approach for breaking the symmetry of microvortices. In addition, manipulating the temperature field with the help of external heat sources, such as strong illumination [69,71,72,73], embedded microheaters [74,75], and heating electrodes [1], can also be used for creating a net flow. Although a common ACET microdevice implements an array of electrode pairs placed at the bottom of a microchannel with a rectangular cross section, more complicated configurations with electrode arrays placed on the top, bottom, and sidewalls of channels with different cross sections have also been studied [69,76,77,78]. Studies with the use of grooves on the channel surface to induce further asymmetry and increase flow have also been addressed, but fabrication of these designs suffers from serious challenges.

Similar to other electrokinetic mechanisms, ACET suffers from some drawbacks, most of which have been addressed to some extent in the literature, as will be shown in this paper. In microfluidic devices, miniaturization can be hindered as the ACET effect originates from the bulk of the fluid and decreasing the channel dimensions can decrease the volume of the fluid flowing inside the channels [3,35]. In addition, ACET depends on the formation of temperature gradients, and therefore, cannot be used with low conductivity fluids. As such, its application in conjunction with DEP, which requires low conductivity fluids for efficient particle sorting, is limited [1,4,5]. Importantly, an excessive temperature rise in fluids with high conductivities can cause the buoyancy force to dominate over the ACET force [4]. The reason is that the ratio of electrothermal force to buoyancy force is proportional to |∇T|/ΔT. Thus, when ΔT>|∇T|, the buoyancy force becomes the dominant force. Finally, increasing the actuation voltage to achieve high fluid velocity can lead to electrochemical reactions which can limit the application of ACET effect on biofluids [1]. There have been some reports on how to mitigate this issue [79].

## 3. Theory

As stated in the previous section, the ACET effect results from the interaction of a non-uniform electric field and a temperature gradient in the bulk of the fluid. The energy balance equation governs the amount of Joule heating as follows [15]:

(1) $$k\nabla^{2}T+\frac{1}{2}‹\sigma {\left|E\right|}^{2}›=0$$

where, *k* and σ are the thermal and electrical conductivities of the fluid, respectively, and ***E*** is the electric field, which can be obtained from the Laplace equation in a homogeneous medium as below:

(2) $$\nabla^{2}V=0$$

where, E=−∇V, and *V* represents the electric voltage.

An order of magnitude estimation of Equation (1) gives [15]:

(3) $$\Delta T\approx \frac{\sigma V^{2}}{k}$$

Based on Equation (3), the temperature rise is directly proportional to the fluid electrical conductivity and actuation voltage squared, which, in most applications, is the control parameter.

The ratio of heat convection to heat conduction in a microchannel is very low (i.e., Peclet ≤0.07) [15,67,80]. Furthermore, it has been numerically shown that, compared to electrical forces, natural convection in micro-channels can be neglected [15,80]. However, for cases with high thermal Peclet numbers, heat convection cannot be neglected [81]. The temperature gradient in the fluid can change the fluid properties, including permittivity ε and conductivity σ, and can be calculated as follows [15]:

(4) $$\nabla \varepsilon =\left(\frac{\partial \varepsilon }{\partial T}\right)\nabla T$$

(5) $$\nabla \sigma =\left(\frac{\partial \sigma }{\partial T}\right)\nabla T$$

In most ACET applications, it is assumed that the rate of change of permittivity and conductivity with the change in temperature is very small [15]. Otherwise, a temperature coefficient needs to be defined to account for the changes in fluid properties [81,82]. As a result of this assumption, the perturbed electric field can be neglected, and the charge convection can be assumed to be much smaller than the charge conduction [15].

The change in electrical properties of the fluid leads to the generation of electrical charge density as follows [15]:

(6) $$\rho_{E}=\nabla \cdot \left(\varepsilon E\right)$$

(7) $$\frac{\partial \rho_{E}}{\partial t}+\nabla \cdot \left(\sigma E\right)=0$$

where, $\rho_{E}$ is the charge density.

Under the effect of the electric field, there is a force applied to the charge density which is [15]:

(8) $$f_{E}=\rho_{E}E-\frac{1}{2}E^{2}\nabla \varepsilon$$

The first term in Equation (8) is the Coulomb force and the second term is the dielectric force.

As charges move in the electric field, they drag the surrounding medium into motion. Therefore, microflows are generated in the fluid and are governed by:

(9) $$\nabla p+\eta {\left|\nabla \right|}^{2}u+f_{E}=0$$

where, η, p, and u are the dynamic viscosity, pressure, and velocity field, respectively. Furthermore, from the conservation of mass for an incompressible fluid, we have:

(10) ∇·u=0

With an order of magnitude estimation from Equation (9), the flow velocity can be written as $\left|u\right|\approx ‹f_{E}›\cdot \frac{l^{2}}{\eta }$, where *l* is the characteristic length of device, which is usually the electrode spacing [4,15,60].

Charge density can be calculated by combining Equations (6) and (7) as follows [83]:

(11) $$\rho_{E}=\frac{\sigma \varepsilon }{\sigma +i\omega \varepsilon }\left(\alpha -\beta \right)\left(\nabla T\cdot E\right)$$

where, ω is the angular frequency of the AC electric field, and:

(12) $$\alpha =\frac{1}{\varepsilon }\left(\frac{\partial \varepsilon }{\partial T}\right)$$

(13) $$\beta =\frac{1}{\sigma }\left(\frac{\partial \sigma }{\partial T}\right)$$

For aqueous solutions and temperatures around 293 K, α and β can be estimated as −0.4% K^−1^ and 2% K^−1^, respectively [84].

With the above approximations, the electrothermal force can be simplified as below [15]:

(14) $$‹F_{E}›=\frac{1}{2}\frac{\varepsilon \left(\alpha -\beta \right)}{1+{\left(\omega \tau \right)}^{2}}\left(\nabla T\cdot E\right)E-\frac{1}{4}\varepsilon \alpha {\left|E\right|}^{2}\nabla T$$

where, $\tau =\frac{\varepsilon }{\sigma }$ is the charge relaxation time of the liquid and is in the range of 0.7–35 ns for conductivities in the range of 0.02–1 S·m^−1^ [41,85]. As stated above, the first term represents the Coulomb force, and the second term is the dielectric force. These forces act in different frequency ranges (i.e., the Coulomb force dominates at low frequencies and dielectric force dominates at high frequencies) and are in different directions [83]. Near a certain frequency, known as the cross-over frequency $f_{c}$, the two forces compete, and flow reversal can occur as a result of switching from a Coulomb force dominant to a dielectric force dominant regime or vice versa [15]. The cross-over frequency can be calculated as below [15]:

(15) $$f_{c}\approx \frac{1}{2\pi \tau }{\left(2\frac{\left|\frac{1}{\sigma }\left(\frac{\partial \sigma }{\partial T}\right)\right|}{\left|\frac{1}{\varepsilon }\left(\frac{\partial \varepsilon }{\partial T}\right)\right|}\right)}^{\frac{1}{2}}$$

For example, the cross over frequency for a biofluid with a conductivity of 1 S·m^−1^ is roughly 200 MHz [61]. ACET force, and thus ACET flow velocity, is higher (up to 11 times) at low frequencies and has no dependence on frequency except for the transition region (i.e., near crossover frequency) [13,41,69].

By taking a closer look at Equation (11), as α, β, and ω are constants, we can conclude that $\rho_{E}\propto \nabla T\cdot E$ [1]. Commonly, in electrokinetics, frequencies much lower than 10 MHz (usually around 200 kHz) are used, where $1+{\left(\omega \tau \right)}^{2}\approx 1$ and dielectric force is negligible (i.e., the Coulomb force is ~11 times larger than the dielectric force) [41]. At these frequencies, there is not enough time for the double layer to form, and thus, the dielectric force is neglected [13]. As a result, the flow direction is determined by the Coulomb force and Equation (14) is reduced to the first term on the right hand side. With a similar argument, as α, β, and ωτ are constants, we can conclude that $\left|‹F_{E}›\right|\propto {\left|E\right|}^{2}\left|\nabla T\right|$, and according to Equation (1), when Joule heating is implemented, $\nabla T\propto E^{2}$, and therefore, $\left|‹F_{E}›\right|\propto {\left|E\right|}^{4}$ [69]. This means that electrothermal force is proportional to the fourth power of the electric field. According to the order of magnitude estimation of velocity obtained from Equation (9), ACET fluid velocity also has a quartic relationship with the actuation voltage. This means that a small increase in the electric field can cause a large increase in electrothermal force, and thus, a significant increase in the resultant flow velocity. As stated above, since electrothermal and electroosmotic flows have similar patterns, one way to distinguish them is to use this proportionality. Since electroosmotic velocity is proportional to the square of voltage, by plotting velocity against applied voltage, the source of microflows can be revealed [14,15].

The theory discussed here is based on an uncoupled model developed by Ramos et al. in 1999 [15]. In such a model, an assumption of a small temperature rise ΔT < 5 K renders the change of fluid properties and electric field with temperature insignificant. However, if the temperature rise is considerably higher, then the fluid properties will change by temperature variations, and therefore, a fully coupled model needs to be used [74,86,87,88]. Hong et al. developed a coupled model, compared it with the classical model, and found that only at small temperature rises do the results of the two models match [87]. More recently, pumping and mixing of non-Newtonian fluids have been studied [89,90,91].

## 4. Electric Field

In lab-on-a-chip applications, the maximum limit for actuation voltage is typically within the range of 5–7 V_rms_, beyond which electrochemical reactions can harm the nature of biofluids. Since, as stated in the previous section, electrothermal force, and thus, fluid velocity is highly dependent upon the applied voltage (i.e., quartic dependence), the increase in velocity of electrothermal based biomedical microdevices is hindered accordingly. To overcome this issue, many studies have proposed and developed new ACET designs with modifications to the electric field, which include either changes to the geometry of the electrode array or introducing asymmetry to the electric potential, using techniques such as travelling wave, multiphase, DC biased, etc. In this section, different strategies proposed for modifying the electric field are reviewed.

### 4.1. Introducing Asymmetry to Geometry

To produce a strong electric field, microelectrodes are typically patterned on the inner surfaces of microchannels [11,66]. Due to changes to the electric field and temperature gradient strengths, by manipulating the electrode configuration, a wide variety of flow patterns and velocities can be achieved [41]. Microelectrode arrays with different shapes have been reported, which can be categorized into two-dimensional (2D) and three-dimensional (3D) geometries [41,85].

**Figure 1:**
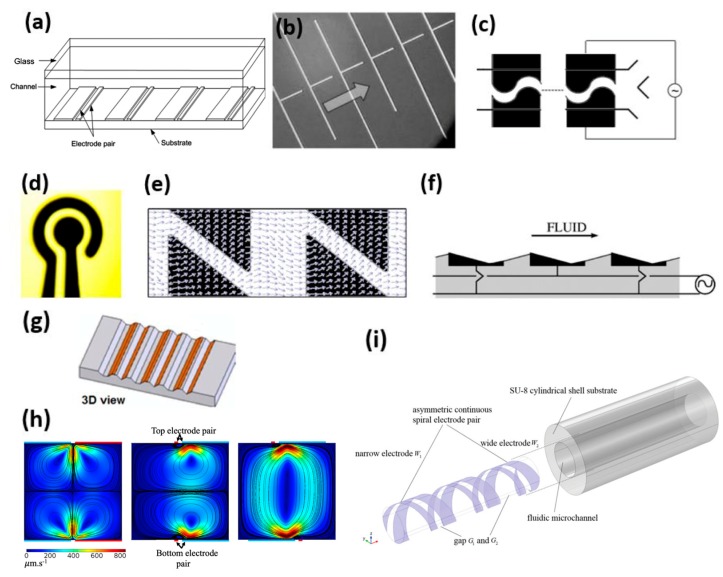
Schematic of different electrode geometries. (**a**–**e**) 2D Electrodes: (**a**) asymmetric rectangular, (**b**) orthogonal, (**c**) meandering, (**d**) concentric, and (**e**) triangular. (**f**–**i**) 3D Electrodes: (**f**) 3D electrode array, (**g**) microgrooved configuration, (**h**) electrodes facing each other, and (**i**) spiral design. Reproduced with permission from [56,60,62,69,77,85,93,94,95].

Due to the simplicity and ease of fabrication, 2D electrode geometries are the most common and can be further categorized into asymmetric rectangular [4,41,69,92], orthogonal [4], meandering [62], concentric [60], and triangular [56], with asymmetric rectangular being investigated the most in the literature (Figure 1a–e). Imposing asymmetry on the electrode pairs induces asymmetry in the resultant microflows, and therefore renders pumping action. Due to this feature of microelectrode configuration, more heat is hypothesized to be generated near the narrow electrode [1]. In general, in such a configuration, the width of the narrow electrode and the gap between electrodes in a pair are the two major design parameters which govern the magnitude of the fluid velocity [41].

Yuan et al. conducted a thorough study on the optimization of 2D rectangular electrode arrays and reported a set of ratios for the corresponding parameters [41]. 2D rectangular asymmetric arrays are used mostly for pumping applications, either alone or with other changes to electric or thermal fields [1,74]. In pumping applications, one or multiple arrays of asymmetric microelectrode pairs are placed perpendicular to the channel length in order to generate a net flow along the channel. In applications, where a lateral fluid mixing is also desirable, however, the electrode array needs to be placed at an angle <90° to the channel length [77,95]. In 2D electrode configurations, by decreasing the gap between electrodes up to a certain point, the resultant fluid velocity can be increased as the strength of the electric field increases. However, if the strength of the electric field is maintained at a constant, increasing the gap between electrodes can enhance the resultant fluid flow, as increasing the gap allows a larger volume of fluid bulk above the gap to experience the strong electric field [41]. One way to enhance the ACET effect is to decrease the width of the narrow electrode, increasing the non-uniformity of the electric field. However, if the narrow electrode is made too small, the strength of the electric field can decrease accordingly, and, as electrothermal force is proportional to the fourth power of the electric field, electrothermal force and, hence, velocity can decrease drastically [41]. As shown, the dimensions and electrode geometry are of great importance to the electrothermal effect. In addition, the channel size and cross section can also significantly affect the resultant flow rate [96]. More on this topic can be found in Section 9. Orthogonal electrode arrays were proposed by Wu et al. for pumping applications with velocities exceeding 1 mm·s^−1^ [4,93]. A meandering electrode array, with a sinusoidal electrode gap, was proposed for rapid mixing of two biofluids [62]. Rapid mixing of high-conductivity (up to 22 S·m^−1^) fluids has been shown using concentric electrode designs [60]. A triangular electrode pattern has also been suggested for micromixing [56].

The backward flow generated above the narrow electrode can negatively impact the use of planar configurations of electrodes for pumping applications, as they cause the average flow in the microchannel to slow down [85]. In 2000, Ajdari, suggested a 3D electrode configuration to circumvent this problem [94] (Figure 1f). Expanding on the same idea, Du and Manoochehri suggested a microgrooved channel (instead of 3D electrodes) to impose spatial asymmetry to the electrode pairs [97] (Figure 1g). More recently, this effect has been numerically simulated with a number of substrate configurations [98]. Manufacturing a channel with modified roughness of substrate surface is easier than manufacturing 3D electrodes. In their work, different shapes of grooves are proposed and experimentally studied. This study showed that by using the microgrooved structure, based on the shape of the grooves, the pumping capacity increases by up to six-fold compared to a planar configuration. In another work, they reported an optimized configuration of such a design [85]. They managed to increase the net flow rate by further suppressing backflows and shortening the streamlines [85]. However, fabrication of such microgrooved structures is more complicated than conventional microchannels with planar electrode design [69]. A variation of such configuration in conjunction with opposing electrodes on the top surface is suggested for mixing applications [89,99]. A similar configuration is also reported where opposing castellated electrodes are utilized to eliminate the ACET vortex and thereby enhance pumping [100].

A two-layer microelectrode configuration, where microelectrodes are facing each other, has been proposed for particle trapping in order to investigate the DEP effect in conjunction with ACET [13]. In this electrode configuration, one electrode is placed on top and the other at the bottom of the channel. A similar configuration is also used for studying Joule heating effects [80], as well as for patterning of colloids when equipped with underlying microheaters [75]. Such an opposing microelectrode configuration with different patterns is also reported for mixing fluids [77,89,101] (Figure 1h). This configuration is also used in rapid electrokinetic patterning, where electrothermal effects play a significant role in particle manipulation [102,103]

More recently, studies have been carried out on utilizing electrodes on all walls of the microchannel in different patterns [104,105]. Using a particular configuration of multiple electrode arrays on side walls, simultaneous mixing and pumping of biofluids in one microchannel during a short time and over a short distance is feasible [104]. In a very recent publication, a spiral electrode pattern has been proposed for simultaneous mixing and pumping which is capable of achieving a velocity of 400 µm·s^−1^ for a biofluid with a conductivity of 0.224 S·m^−1^ (Figure 1i) [95].

### 4.2. Introducing Asymmetry in Electric Potential

In addition to an asymmetric electrode geometry, the electric field can also be modified to enhance the electrothermal effect. Applying a DC potential to the electrode pairs in addition to the existing AC potential, using a multiphase AC signal in the form of a travelling wave, and utilizing a two-phase actuation system are examples of such modifications, each of which is briefly described here.

#### 4.2.1. DC Biased

**Figure 2:**
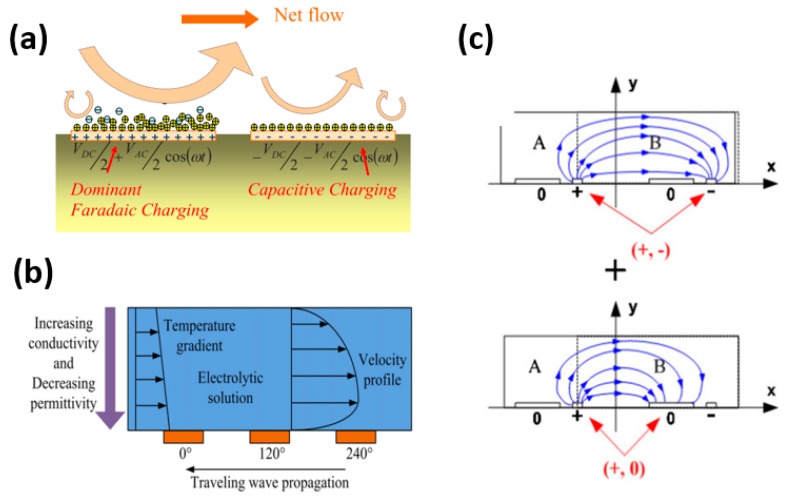
Schematic of different mechanisms for imposing asymmetry to the electric field in alternating current electrothermal effects (ACET) devices. (**a**) Direct current (DC) biased configuration. The left electrode is always at a positive potential and subject to faradaic charging, while the right electrode is always at a negative potential and subject to capacitive charging. (**b**) Travelling wave configuration. The induced temperature gradient generates a charge density which is moved with the travelling electric field. Note that the direction of propagation of travelling wave is opposite to the direction of fluid flow. (**c**) Two-phase asymmetric configuration. The narrow electrodes in two adjacent pairs have different polarities. The superposition of the two configurations enhances the electric field in region B. Reproduced with permission from [6,68,69].

In this method, symmetric electrodes are used but one electrode is always at a positive potential and the other is always at a negative potential subject to faradaic charging and capacitive charging, respectively (Figure 2a) [6]. Hence, positive charges on both electrodes lead to unidirectional flow. The advantage of this configuration is that pumping action can be achieved by a symmetric pair of electrodes.

#### 4.2.2. Travelling Wave (TW)

For the first time in 1966, Melcher observed pumping of fluids with very small conductivities, by imposing a temperature gradient along the depth of the channel in conjunction with travelling wave induction [64]. In 1967, Melcher and Firebaugh further investigated the phenomenon and presented the governing equations [65].

Fuhr et al. studied a travelling wave induced micropump and modified the principles presented by Melcher and Firebaugh [7,65]. Later, in 1994, the same group explained the mechanisms in which temperature gradients (both externally applied and internally generated) are used in conjunction with a travelling wave [66]. With an induced temperature gradient in the fluid bulk, free charges can be generated, and the travelling wave can move them along the channel above the microelectrodes causing fluid flow. This principle is further illustrated in Figure 2b. The mechanism of applying a temperature gradient in this design can be external (i.e., bed heating) or self-generated (i.e., Joule heating).

In 1992, Fuhr et al. experimentally investigated a high frequency travelling wave induced micropump with self-generated temperature gradient, i.e., higher temperatures at the bottom of the channel in the space between electrodes and lower temperatures at the top of the channel [7]. In this case, the resultant fluid flow is in the opposite direction of the travelling wave, as also verified by Liu et al., [68]. Their measurements of flow velocity show a quadratic relationship with voltage caused by applying an external temperature gradient [13,106]. Following the study of Fuhr et al., several numerical investigations of travelling wave induced electrothermal flow were also carried out [68,107].

#### 4.2.3. Two-Phase Actuation

In 2011, Zhang et al. proposed a two-phase planar asymmetric electrode design which yielded an increase of 25–50% in flow rate compared to conventional single-phase asymmetric configurations [69]. In this design, an AC signal of 0°/180° was applied to the narrow electrode in an asymmetric electrode pair (Figure 2c). This led to an enhanced electric field. As the temperature gradient has a quadratic relationship with the electric field, temperature, and therefore, flow rate increased accordingly.

## 5. Temperature Field

### Internal and External Heating

In an ACET device, heating of the fluid is the source of thermal gradients which can eventually lead to electrothermal microflows. Heating can be either internal (i.e., Joule heating) or external. 

Internal heating refers to Joule heating of the fluid upon activation of the electric field. In a rectangular asymmetric electrode configuration, the temperature rise above the narrow electrode is higher than that above the wide electrode. Joule heating scales with conductivity of the fluid and applied voltage (Equation (1)). As a result, fluids with low conductivity need a higher electric field to produce sufficient thermal gradients. However, at high applied voltages, electrochemical reactions can occur, which can damage the fluid and electrodes. This issue can be resolved by applying an external heat source and keeping the applied voltage low.

A few methods have been proposed for external heating, including strong illumination [69,71,73], integrated heating elements (ohmic heating element) [39], heating of the electrodes [1], and thin film resistive heaters [74], the latter of which is shown to be portable and more efficient [74,75]. 

Incorporation of external heating allows the electrothermal flow to be controlled independently of the electric field strength [39,74]. Therefore, the effect of fluid properties on the resultant flow is minimized [39], and manipulation of low conductivity fluids with the electrothermal effect becomes feasible [74]. In addition, by means of external heating, the electrothermal flow can be implemented in conjunction with other electrokinetic methodologies, which work under a uniform electric field, such as electrophoresis and electroosmosis [74,75]. External heating strategies also enable control of the direction of fluid flow [1,71,106].

Green et al. performed a thorough study on strong illumination as an external heat source [71,106]. The resultant velocity field in the case of external heating can be obtained from the equation below [13,106]:

(16) $$\left|u\right|\approx 3\times 10^{-3}\left(\frac{\varepsilon V^{2}}{\eta }\right)\left|\frac{\partial T}{\partial y}\right|\left(\frac{1}{\sigma }\right)$$

where, $\frac{\partial T}{\partial y}$ is the external thermal gradient along axis y. Unlike Joule heating, here the velocity has a quadratic relationship with voltage, which was verified experimentally by Stubbe et al., where ohmic heating elements were used [39].

Depending on the amount of heat flux introduced into the fluid bulk by the external heating, the effect of voltage on the flow velocity changes. In 2013, Yuan et al. found that if the heat flux is small (around 10^4^ W·m^−2^), it can be counteracted by the Joule heating of the fluid, and the relationship between fluid velocity and voltage can be between quartic and quadratic [1]. However, if the heat flux is relatively large and the fluid is mostly under the influence of external heating, the fluid velocity will have a quadratic dependence on the voltage.

Furthermore, thin film microheaters can be embedded in the substrate below the electrodes, separated by an insulating layer [75]. Such a heating mechanism can yield a thermal efficiency of close to 100% [74]. Velasco and Williams showed the application of thin film heaters for assembly of colloids [75]. The geometry of the assembly is governed by the geometry of the array of microheaters. Therefore, particles can be trapped in a larger area when compared to conventional particle trapping techniques [13]. The assembly of 1 µm and 2 µm particles was achieved in the frequency range of 1–200 kHz, while no particle aggregation was observed at other frequencies. Since the temperature gradient is the underlying mechanism, microheaters cannot be patterned close to each other. Furthermore, with thin film microheaters, sharper temperature gradients exist near the electrodes which result in a larger value of $\nabla T\cdot E^{2}$ [74]. Compared to Joule heating, thin film microheaters alone require only 40% of the power to produce the same flow rate. Without a change in power, the flow rate can be increased to 250% of that of solely Joule heating [74]. Both the proximity of thin film resistive heaters to each other and their distance to the regions of maximum electric field strength determine the resultant flow regime [74,75]. The maxima of temperature gradient and electric field need to occur in the same region in the bulk of the fluid in order to maximize the product of $\nabla T\cdot E^{2}$ [74]. More recently, Williams and Green applied the same idea to a symmetric pair of electrodes, which is more desirable for DEP applications, and carried out numerical simulations towards finding an optimum location for the heater with respect to the electrodes [108]. 

In spite of a fundamental factor for generation of electrothermal flow, Joule heating can be an unwanted effect in other electrokinetic devices [80]. For example, in a DEP based particle manipulation device, Joule heating can harm the biological fluid and form electrothermal microflows taking particles away from their intended spot of sorting or trapping [80,81]. For this reason, studies have been conducted to gain a better insight into Joule heating [80] and its effects on insulator-based DEP devices [81,109].

Almost all electrothermal designs studied in the literature are checked for excess temperature rise due to Joule heating, as this can negatively impact their potential application with biofluids. For example, Du and Manoochehri compared the Joule heating in their proposed microgrooved and planar configurations at a wide range of conductivities [85] and found no major difference between the two structures. In devices with thin film microheaters, temperature rise is shown to be half of that of Joule heating, which further corroborates the high efficiency of these devices [74].

Similar to Joule heating, external heating may not always lead to an enhanced electrothermal effect. Zhang et al. studied the effect of strong illumination coupled with their two-phase planar electrode configuration [69]. Interestingly, they observed a decrease in velocity when a strong illumination was applied. This observation was justified by assuming that the flow direction generated by the illumination is opposite to that generated by Joule heating [83]. As mentioned earlier, for external heating to be effective, heat flux introduced into the system must be significantly higher than the Joule heating generated by the system. Not clearly stated by Williams [74], however, is that their reported results show that by increasing the conductivity, the ratio of electrothermal force generated by thin film heaters to that generated by Joule heating gradually decreases. This indicates that the coupled electric and temperature fields need to be solved for higher temperature rises (>5 K) [82,86].

ACET flow velocity has a strong dependence on the applied voltage ($u\propto \;V^{4}$, when no external heating is applied). However, voltage can be increased up to a certain point, usually below 7 V_rms_, to avoid thermal damage to the biofluid [1,11,14,41,60,71,75]. To solve this problem, Yuan et al. introduced a thermally biased configuration, where one electrode in a pair is at a higher temperature than the other one [1]. In this way, the temperature gradient in the fluid can be controlled independently from the voltage.

At frequencies below 10 MHz, where the Coulomb force is the dominant force, the electrothermal force can be simplified to $F_{E}=\rho_{E}E$. Since, due to the limitations discussed above, there is an upper limit for the applied electric field, the electrothermal force can be increased further by increasing the charge density, $\rho_{E}$. As $\rho_{E}\propto \nabla T\cdot E$, Yuan et al. attempted to increase the electrothermal force by increasing the temperature gradient by means other than Joule heating [1]. In their design, unidirectional flow was obtained with symmetric electrodes, making it possible for their configuration to be used either as a micromixer or a micropump depending on the applied voltage, frequency, and heat flux. When imposing external heat flux to the narrow electrode in an asymmetric pair, they obtained a velocity 5.7 times higher than that of a regular asymmetric ACET micropump. Their simulation results show that by applying the heat flux to the wide electrode, the direction of the flow above the electrodes can be reversed. This finding corroborates their hypothesis that the generated heat on the narrow electrode is the primary cause of pumping in a conventional ACET device, and that external heating has a strong influence on the pumping performance.

## 6. Fluid Flow Regime

### 6.1. Flow Velocity

Almost all the work that has been performed dealing with the electrothermal effect is an attempt to increase the ACET velocity in high conductivity fluids while avoiding significant increase in temperature and voltage. Many studies have attempted to increase the electrothermal flow velocity by focusing on the governing parameters mentioned in Equation (14) and have proposed new designs to increase the velocity based on their interpretations of the formula. The maximum of the flow velocity reported in these studies differs both in its magnitude and location (i.e., height/distance from channel bottom/electrodes).

Since electrothermal velocity has a quadratic relationship with the temperature gradient, many studies have focused on increasing this parameter of electrothermal force to increase the flow velocity. External heating has been proposed and thoroughly investigated for this purpose, which is discussed in Section 5. By simplifying the equation of electrothermal force to $\left|‹F_{E}›\right|=\xi \left(\omega \right){\left|E\right|}^{2}\left|\nabla T\right|$, Zhang et al. concluded that by increasing both the strength of electric field and the temperature gradient, a significant increase in electrothermal force and thus fluid velocity could be obtained [69]. Taking advantage of this combination, by developing a two-phase asymmetric planar electrode design in which a stronger electric field is obtained, they demonstrated flow velocities reaching 25–50% higher than those of conventional single-phase configurations. In this simplified equation, ξ(ω) is a function of frequency and the angle between the vectors of applied field and temperature gradient. They also found that fluid velocity is much higher at low frequencies, which was also verified by Williams who determined that pumping rates at high frequencies (>$f_{c}$) drop to 10% of that at low frequencies [74].

To accurately measure the electrothermal velocity field, usually micro particle image velocimetry (micro-PIV) is used. The assumption of this method is that particles follow the fluid flow and are not under the influence of any other forces [110,111,112]. Therefore, ACET velocity needs to be measured at a height with a negligible DEP effect on particles [1]. At a distance close to electrode surface, the DEP effect on particles is significantly high, which can cause them to be trapped at the electrode edges [1]. As a result, depending on the channel height and size of tracer particles, ACET velocity is typically measured at a height of 10–50 µm above the electrode surface. A good control for this effect is carried out by Wu et al., where, they found, by using particles with a size of ~500 nm, an order of magnitude estimation yields that at ~5 V_rms_ particles move with a DEP-induced flow velocity of 0.11 µm·s^−1^ at the height of 10 µm above the electrodes [4]. Since ACET velocity is often higher than 100 µm·s^-1^, DEP velocity is negligible at this height. The highest electrothermal velocities reported are taken at a height of 20–50 µm above the electrodes [1,13,92]. The lowest electrothermal velocity occurs very close to the electrodes (~5 µm above electrodes in a microchannel with a height of 200 µm) [92]. These observations are in agreement with the physics of the electrothermal effect as it is created in the bulk of the fluid.

Increasing the number of microelectrode pairs has shown to result in increasing ACET fluid flow of high conductivity biofluids at voltages above 4 V_rms_ [92]. In addition, flow velocities measured experimentally are typically smaller than those predicted numerically, sometimes by orders of magnitude, which can be justified to be related to the experimental conditions. Here, we discuss some of the experimental issues reported in the literature. 

In their experimental study, Sigurdson et al. showed an electrothermal flow velocity of 100 µm·s^−1^, which was reported at a lower voltage in a numerical study [14]. Since their simulations are performed for a 2D geometry, they attributed this discrepancy to neglecting the out of plane heat transfer, which is significant in devices with a small channel height (i.e., ~200 µm). In their later work, since they encountered the same issue (i.e., observing an experimental velocity of 1.5 orders of magnitude lower than the numerical result), they defined an effective voltage [61]. For this matter, a reduction coefficient of 0.38 was introduced to correct the actuation voltage in the numerical simulations. With the modified voltage, the velocity–voltage curve of numerical study closely matches the corresponding curve of the experimental study.

Studying the Joule heating effect, Williams et al. [80] defined a coefficient, *E_rel_*, for medium conductivity, to account for the loss in applied electric field, which they attribute to the electrical resistance of their electrode material (i.e., indium tin oxide (ITO)). Therefore, by using a more conductive material for electrodes (e.g., gold), there will be less potential loss (i.e., larger value of *E_rel_*). Sin et al. showed a significant deviation of experimentally measured fluid velocity from the theoretically predicted one for fluids with conductivities on the order of 22 S·m^−1^ [60]. The reason for this discrepancy was assumed to be the deterioration of the electrode surface due to electrochemical reactions at high conductivities, which is not accounted for in the numerical model. The same argument is also reported as the reason for deviation of experimental data from simulation in other studies [76,105]. With the surface of the electrode being altered, the electric field, flow patterns, and velocities are no longer predictable as the actual electric potential can be significantly decreased [76]. In addition to high conductivity, increasing voltage above 5.5 V_rms_ is also reported to cause electrode deterioration [76]. Evaporation, and thus a change of the medium’s properties, is also mentioned as another possible factor for this discrepancy. In addition, the buoyancy effect can play a role in causing this discrepancy, since a high conductivity medium can experience high temperatures. The ratio of the electrothermal force $F_{E}$ to buoyancy force $F_{B}$ can be approximated as the following [4]:

(17) $$\left|\frac{F_{E}}{F_{B}}\right|=7.95\times 10^{-12}\left(\frac{\nabla T}{\Delta T}\right)\cdot E_{rms}^{2}$$

As suggested by this approximation, for buoyancy to be negligible, temperature gradient ∇T needs to be much higher than temperature rise ΔT. As a result, for enhancing the ACET performance, device elements (e.g., electrodes) with relatively high thermal conductivities need to be implemented [4].

### 6.2. Direction of AC Electrothermal (ACET) Flow

In micropumps with planar electrode configurations, two microvortices on both electrodes in every pair are in competition to determine the direction of net flow [85]. In conventional rectangular asymmetric configurations, since the fluid flow spends more time on the wider electrode, the direction of ACET net flow is determined by the microvortices on the wider electrode [4].

It has been experimentally shown that the direction of flow can be controlled by external heating, i.e., switching the heat flux between wide and narrow electrodes [1,71,106]. This is true for both rectangular symmetric and asymmetric electrode configurations. Additionally, some studies have been performed on the use of unique device configurations to easily toggle the direction of net flow and introduce mixing [113,114], however, these models have not yet been rigorously validated.

### 6.3. Flow Reversal

One major advantage of ACET is that its velocity is generally independent from frequency, however, at around the crossover frequency, flow reversal occurs, and thus, lower velocities (~10–20% of velocity of low frequencies) are generated [39,69,74,115].

Flow reversal has also been observed in devices with external heating. In 2014, Liu et al. numerically investigated applying a temperature gradient along the channel length on rectangular symmetric electrode arrays and compared it to the conventional asymmetric array [68]. They observed unidirectional pumping in the direction from higher to lower temperatures at 100 kHz (i.e., from the narrow electrode to wide electrode). They also observed that by increasing the frequency to 500 kHz, the direction of flow in a symmetric array reverses but still maintains a unidirectional flow, whereas, in the asymmetric array, the unidirectional flow turns to vortices with no pumping action. 

In general, factors other than frequency can also lead to flow reversal. For example, ACEO systems can face flow reversal at higher voltages due to faradaic charging [116]. Transition between ACEO and ACET mechanisms [93] and steric effect can also be important [117,118]. However, none of these reasons can justify the flow reversal in the DC biased AC electrothermal device of Lian et al. [6], which is still open for further investigation.

Flow reversal is considered by most studies as a disadvantage in ACEK due to the uncontrollable and unpredictable nature of this phenomenon. However, some studies have investigated controllable flow reversal by tuning the actuation frequency, switching electric field, and/or applying external heat sources [1,39,68,113,114].

## 7. Application

Most common applications of the electrothermal effect in micro systems include mixing and pumping of fluids and particle manipulations [1,13,60,62]. A good example of using the electrothermal effect for mixing is immunoassays [11,14,20,61,119,120,121]. Immunoassays are biochemical tests in which the concentration of macromolecules (e.g., ligands or proteins) in a biofluid is measured by the use of an antibody. The antibody is immobilized on a surface and then the biofluid of interest is introduced above the surface. The concentration of macromolecules is measured by the number of macromolecules attached to the antibodies. The key factor in this process is for macromolecules to contact the antibodies repeatedly so that all possible bindings take place and lead to a precise sensing result. Since this process is diffusion limited, incubation time can take hours [61]. By using the electrothermal effect, the chance of macromolecules reaching the sensing area can be increased, thereby significantly decreasing the response time, enhancing the bindings by seven to nine times within minutes [14,61]. Sigurdson et al. investigated this idea numerically and found that by applying a voltage of 6 V_rms_, an increase of seven times in the amount of bound antigen can be reached. It should be noted that electrothermal stirring is effective only when Damkohler ≥100, i.e., when the process is diffusion limited and not reaction limited. Therefore, electrothermal stirring is not significantly effective for reaction limited systems such as DNA systems. Sigurdson et al. also found that the electrothermal stirring is especially effective in the space above the electrode gap, where the velocity and the concentration gradient are high, making it the optimum place for antibodies to be immobilized [11,14,61]. This was proven experimentally in their later work [61]. Huang et al. found the optimum reaction site to be closer to the negative electrode in a symmetric rectangular pair [122]. In this case, both Damkohler and Peclet numbers need to be considered. Electrothermal stirring is best for mass transport limited regimes, where the Peclet number is relatively low, and convection contributes more to the overall mass transport than diffusion. Therefore, by utilizing the electrothermal effect, the required sample volume can be reduced, leading to higher efficiency devices [14]. It has also been shown that in immunoassay applications, electrothermal force can be a better choice compared to electroosmotic force, as electroosmotic force may cause the antigen–antibody bounds to fall apart [14].

In 2012, Sasaki et al. used a meandering electrode configuration in a Y-shaped channel to mix two high salt content fluids [62]. They reported a fivefold reduction in mixing time compared to diffusional mixing. In their study, the dependence of mixing index on salt concentration, frequency, and mixing time was investigated. It was concluded that the meandering structure was suitable for salt concentrations of 10^−3^ to 10^−1^ mol·dm^−3^, provided that the frequency lied in the range of 100–200 kHz, which is typical for electrothermal devices. A long range ACET effect, where centimeter scale vortices are generated, can also be used for mixing purposes [70].

Further development on electrothermal based immunoassays was carried out by Liu et al. in 2011 [11]. They decreased the incubation time from 30 min to 3 min by implementing ACET in their immunoassay with the conventional asymmetric electrode array. A study on electrode geometry on capacitive immunoassays showed that electrode geometry is an important component in high electric field configurations with asymmetric geometries rendering higher detection efficiency [123]. Another study suggested that the placement of electrodes on the same surface as the reaction site renders the most efficient configuration for immunoassays [121]. Selmi et al. studied the effect of temperature on immunoassays with asymmetrical electrodes [124]. The use of a pulsed ACET flow and amplitude modulated (AM) sine waves has also been reported for enhancing mixing efficiency in immunoassays [125,126].

Electrothermal effect in conjunction with dielectrophoresis can be used for the trapping and assembly of particles, patterning of colloids, and preconcentration of biological samples for detection and characterization purposes [13,56,59,75,127,128,129,130,131]. Additional studies have shown the enhancement of DNA hybridization with ACET configurations [132]. Recently, the concept of an AC electrothermal micropump was applied to a cell-culture system (with culture media of conductivities up to 2 S·m^−1^) towards the development of organ-on-a-chip and human-on-a-chip systems [63,133].

Recent studies have also shown that, although other ACEK effects such as DEP can enhance trapping of biological samples, they render nonspecific responses [134].

## 8. Substrate Material

As electrodes are patterned on a substrate, and the substrate is one of the channel walls through which the heat is dissipated, the choice of substrate material is of great importance [135]. The materials typically used for the fabrication of microfluidic devices include silicon, glass, polydimethylsiloxane (PDMS), and polymethylmethacrylate (PMMA). As a commonly used substrate material, glass has a lower thermal conductivity compared to silicon and can yield higher temperature gradients, and thus, higher rates of electrothermal flow [105]. Heat transfer through the substrate can be controlled by other means too. For example, a thermoelectric cooler under the silicon substrate has been suggested for maintaining the temperature gradient of interest in an ACET device [61]. As opposed to glass, silicon has a higher heat transfer coefficient, and thus, is a good candidate for applications where excessive temperature rise is undesirable [61,85,87,92,136]. Silicon is also admired for its precise geometrical features and low surface roughness [85]. Compared to silicon, the thermal conductivity of PDMS is much lower, causing the temperature rise in PDMS microchannels to be significantly high [92,136].

## 9. Channel Height

Unlike ACEO, the origin of ACET is the charge density generated in the bulk of the fluid. Therefore, the height of the microfluidic channel plays an important role in the formation of microflows. The microflows will be suppressed when the channel height is too small (<200 µm in a typical microfluidic ACET device) [13,76]. In contrast, ACEO devices utilize a thin layer of electric double layer responsible for dragging the fluid bulk through the microchannel, meaning that reducing the channel height causes the ACEO effect to be more effective. 

It has been shown that in two-phase actuation systems, the optimal channel heights are in the range of 500–1000 µm [69]. In a planar configuration, the maximum velocity is reached at the height of ~500 µm, above which no significant difference in the flow rate can be observed [76,85]. In microgrooved structures, however, it is shown that a velocity of five times higher than planar electrode configurations can be reached at a significantly smaller channel height, i.e., ~50 µm, which is 10% of the optimal height in conventional and two-phase systems. This feature of the microgrooved configuration helps further miniaturizing the ACET based devices. As mentioned above, miniaturization is hindered in ACET devices due to dependence of the microflows on the channel height. With increasing the channel height above 50 µm in a microgrooved configuration, the velocity drops but still stays at higher values compared to a planar configuration.

In the case of patterning electrodes both on the top and bottom of the microchannel in an ACET device, increasing the channel height above 200 µm causes the velocity profile to become more similar to that of an ACEO device [104]. A thorough study on the effect of channel height in micropumps can be found in reference [76].

## 10. Numerical and Experimental Settings

In this section, some key points and influential factors in numerical simulation and experimental setup of ACET devices are briefly reviewed. To assure that the same conditions are valid to apply to other devices of interest, the readers are advised to refer to the articles associated with each statement.

### 10.1. Numerical Simulation

- Since the wavelength of the electric field is typically larger than the dimensions of the microchannel, electrostatic assumptions can be made [41].
- If electrodes are thin, they do not affect heat transfer [61]. Sufficiently thin electrodes (e.g., ~1200 Å thick) can be assumed isothermal [13,106].
- In typical ACET devices, the ratio of buoyancy force to electrothermal force, i.e., $\left|\frac{F_{B}}{F_{E}}\right|$ , is estimated to be in the range of $7\times 10^{-4}-27\times 10^{-4}$ [41,85]. When buoyancy is included in the simulations, only a 0.1–0.8% decrease in flow velocity is obtained [41]. Therefore, the buoyancy effect can be neglected in simulations. However, at large length scales and low voltages, it becomes important [41].
- ACEO effect can be neglected at high frequencies and high conductivities [41].
- 2D and 3D simulations usually render the same results. For example, Yuan et al. [41] reported a 1.48% difference in velocities obtained from their 2D and 3D simulations. Therefore, 2D simulations can help saving computational time [41].
- While in most studies electric and thermal fields are considered independent, using the results of Loire et al. [86], Williams [74] conducted numerical simulations with coupled electrical and thermal fields as $\nabla^{2}V=\gamma \cdot \nabla V$, where γ=−β∇T, instead of the conventional sequential method i.e., $\nabla^{2}V=0$. It was shown that when the temperature rise in a system is >5 K, the two fields can no longer be considered independent [86].
- New methods based on Lattice Boltzman were reported for studying ACET flows [137,138].

### 10.2. Experimental Setup

- The lighting on the microscope, on which the ACET device is mounted, can play as an external heat source and interfere with the experiments, and thus cause unreliable results. In order to reduce the effects of microscope light, either it needs to be set at its lowest power [13] or a heat absorbing filter between the device and the objective lens needs to be used [62]. Otherwise, illumination effects must be taken into account as an external heat source.
- To reduce the effect of Brownian motion, the average of at least four velocity readings at each voltage setting is recommended to be taken [13].
- If the work involves study of temperature on DEP effect, a non-invasive method (i.e., with no particles involved) must be used to measure temperature in the device. Laser-induced fluorescence (LIF) thermometry, in which a dye is used to measure temperature, is recommended for this purpose [80].
- For measuring velocity, to ensure repeatability, particles must be tracked over at least three pairs of electrodes along the microchannel [41,76].
- For generating effective electric field at the electrode surface, electrodes should be fabricated relatively thin, e.g., 50–100 nm [32].

## 11. Future Work

Although utilizing the ACET effect for various biomedical applications has been investigated extensively over the past two decades, more investigative work is yet to be carried out to further our knowledge of the phenomenon. Existing drawbacks must be better addressed in order to facilitate utilization of this effect in biomedical laboratory settings. For example, temperature rise, and its detrimental effects on biological samples, is of great concern while using the ACET effect in microfluidic devices. Substrate materials with high heat conductivities, which enable a low temperature rise while keeping high voltages, must be investigated to achieve strong electrothermal flow.

While plenty of novel and efficient electrode designs have been proposed for pumping and mixing applications, the majority of such reports are numerical studies. A lack of experimental studies that would reveal the hidden challenges in applying these novel strategies to real life applications exists in the literature. Such lack of experimental works is mainly due to the limitations in microscale fabrication and electrode degradation occurred at high voltages. To address such fabrication challenges, techniques for low cost fabrication of prototypes of such complicated designs and investigation of different electrode materials and coatings to withstand high voltages [79] are in great demand.

## Appendix A

The majority of the publications cited throughout the manuscript which have made major contributions are summarized in Table A1.

**Table A1:** A short summary of relevant articles studying the electrothermal effect, useful for new researchers in this field as a guide to what has been done and those who are working in the field.

Article	Application	Achievement	Specific Observations
[71]	Mixing	Experimental study of illumination-induced electrothermal	The direction of force at high frequencies is form hot regions to cold regions while at low frequencies the opposite is true.
[14,61]	Mixing	Increasing the binding rate and significantly decreasing the incubation time to minutes	Binding rate increased by a factor of nine compared to diffusion-limited reaction
[4]	Pumping	Study of pumping for two electrode configurations of planar asymmetric and orthogonal	Orthogonal configuration yields higher velocities
[13]	Particle manipulation and pumping	Manipulation of particles and fluids of high conductivity at low voltages using a parallel plate and a planar asymmetric electrode configuration	Velocity of 162 µm·s^−1^
[93]	Pumping	Numerical and experimental investigation of flow reversal in orthogonal electrodes	Change of flow patterns is a result of change from alternating current electrothermal (ACET) effects to alternating current electroosmosis (ACEO) phenomenon
[6]	Pumping	Applying asymmetry in electric potentials in conjunction with spatial asymmetry	Velocity of 2500 µm·s^−1^
[62]	Mixing	Introducing meandering electrode configuration with electrothermal effect in a Y-shaped channel	Fivefold reduction of the mixing time of high salt content fluids compared to diffusion-limited methods
[97,85]	Pumping	Introducing microgrooved electrode configuration	Five times increase in pumping rate compared to conventional planar configurations
[69]	Pumping	Introducing two-phase AC signal configuration	25–50% faster flow rates in two-phase configuration compared to the conventional single-phase configuration
[60]	Mixing	Introducing concentric electrode design	Velocity of 70 µm·s^−1^
[11]	Mixing	Using asymmetric electrodes for immunoassay	Ten times acceleration in binding rate compared to diffusion-limited method (30 min vs. 3 min)
[1]	Pumping	Thermally biased ACET pumping using symmetric and asymmetric electrodes	Velocity of 750 µm·s^−1^
[75]	Particle manipulation	Using parallel plate (opposing) electrodes in conjunction with thin film resistive heaters	Sorting between 1 µm and 2 µm particles
[92]	Pumping	Study on the effect of the number of electrode pairs over channel length; asymmetric planar electrodes	Increasing the number of electrode pairs helps increase the pumping efficiency
[105]	Pumping	Introducing electrodes both on top and bottom of the microchannel; asymmetric planar electrodes	Opposing electrodes increase the flow rate by 105%
[76]	Pumping	Multiple Array Electrothermal Micropump (MAET) with different actuation patterns and cross sections	Flow rate of 16 × 10^6^ µm^3^·s^−1^
[96]	Pumping	3D circular electrodes	Flow rate of 15 × 10^6 ^µm^3^·s^−1^
[104]	Mixing and pumping	Numerical investigation of simultaneous pumping and mixing by introducing microelectrodes on side walls of the microchannel	Mixing efficiency of 80% in ˂3 min and over a length of ˂600 µm
[135]	Pumping	Numerical study of multiple array ACET channel	Flow rate of 16 × 10^6 ^µm^3^·s^−1^
[74,108]	Pumping	Study of using thin film heaters for pumping	2.5 times faster flow rate with thin film heaters compared to Joule heating alone
[63]	Pumping	Application of ACET pumping to cell culture on chip	Flow rate of 44.82 µL·h^−1^
[139]	Particle manipulation	Combining ACET and dielectrophoresis (DEP) for detection of circulating cell-free DNA (cfDNA)	Detection of cfDNA in 10 min in concentrations as low as 43 ng·mL^−1^
[140]	Pumping	Numerical and experimental study of the effects of conductivity and channel height on ACET flow	A critical conductivity exists below which there is no net flow and there exists only microvortices
[119]	Mixing	Quantum dot-linked immunodiagnostic assay coupled with ACET mixing	Reduction of detection time from 3.5 h to 30 min using a volume of 2 µL
[59]	Particle manipulation	Development of a mathematical model for rapid electrokinetic patterning (REP) REP based on ACET and DEP	Increasing particle size results in an increase in ratio of ACET to DEP velocity and therefore results in a lower focusing performance
[73]	Mixing	Experimental study of light actuated ACET flow	When AC frequency is above liquid charge relaxation frequency, natural convection is above 35% of the ET flow.
[123]	Mixing	Numerical and experimental comparison of immunoassay performance when using symmetric or asymmetric electrodes	Symmetric and asymmetric geometries render different performance efficiencies only at high electric fields
[102]	Particle manipulation	Numerical and experimental study of electrode material in REP	Titanium electrodes are more efficient than conventionally used indium tin oxide (ITO) electrodes
[141]	Mixing	Numerical and experimental study of AC biased concentric electrodes in biosensors	Faster sensing speed compared to diffusion-limited conditions
[142]	Mixing	Numerical and experimental study of rotating asymmetric electrode pair; Supplying controlled drug concentration to tumor cells	Mixing efficiency 89.12%
[70]	Mixing	Numerical and experimental study of long-range fluid motion induced by ACET microvortices	Centimeter scale ACET vortices are observed
[124]	Mixing	Numerical study of the effect of temperature on binding efficiency in immunoassays	Keeping external surfaces of the microchannel at a constant temperature improves the binding efficiency
[143]	Mixing	Numerical and experimental-3D electrodes embedded inside walls of the channel	Mixing efficiency of 90%
[113]	Pumping	Numerical and experimental study of bi-directional micropump using asymmetric planar electrodes	1500 µm·s^−1^ fluid velocity
[121]	Mixing	Numerical study of electrothermal effect in immunoassays	Placement of electrodes on the same wall as the reaction surface renders the best performance of the biosensor
[126]	Mixing	Study of pulsed ACET flow for detection of dilute samples of small molecules	83% mixing efficiency over a length of 400 µm
[125]	Mixing	Numerical investigation of amplitude modulated (AM) sinewave	100% mixing efficiency with maximum 5.5 K temperature rise
[144]	Mixing	Numerical investigation of the effect of ionic strength on mixing	Mixing efficiency 90%
[134]	Pumping	Experimental study of an immunoassay chip featuring an ACET micropump	Reducing incubation time to 1 min vs. hours in conventional methods
[99]	Simultaneous pumping and mixing	Numerical study of high throughput mixing using opposing asymmetric microgrooved electrodes and symmetric electrode pair	Mixing efficiency of 97.25%
[114]	Simultaneous pumping and mixing	Numerical study of bi-directional pumping and mixing by switching electric potential on planar electrodes	Mixing efficiency of 90% Pumping velocity 90 µm·s^−1^
[90]	pumping	Numerical investigation of pumping non-Newtonian blood flow	Velocity of 0.02 m·s^−1^
[89]	Mixing	Numerical investigation of the effect of shear dependent viscosity on mixing efficiency and flow rate using opposing asymmetric microgrooved electrodes and symmetric electrode pair	In similar configurations, dilatant fluids show better mixing efficiency compared to pseudoplastic fluids
[101]	Mixing	Study of arc electrodes in ring-shaped microchamber	100% mixing efficiency at 8 V
[127]	Trapping	Using ACET and DEP to preconcentrate and detect E. Coli	Method can detect concentrations two orders of magnitude smaller than what is possible with diffusion limited methods
[133]	Pumping	Using laser etching on ITO glass to pattern electrodes for pumping cell culture medium in a 3D biomimetic liver lobule model	2 µm·s^−1^ at 5.5 V
[100]	Pumping	Using castellated electrodes; combined DEP and ACET EHD for bioparticle delivery	Negative DEP prevents particles from colliding with channel surfaces; castellated electrodes eliminate ACET vortices
[138]	Pumping	Combining ACET and negative DEP for long range cell transport and suspension in high conductivity medium	DEP is essential for cell suspension under ACET effect
[95]	Simultaneous pumping and mixing	Numerical investigation of 3D asymmetric spiral microelectrode pair	Flow rate 440 µm·s^−1^
[91]	Pumping	Numerical investigation of the effect of electrode configuration on pumping mechanism of non-Newtonian blood flow	Ring shaped electrodes are the optimal configuration for blood flow pumping
[88]	Pumping, mixing, and trapping	Study of 3D particle-fluid flow under simultaneous effects of ACET, thermal buoyancy (TB), and DEP using multi-layered electrodes	Long range vortices induced by ACET and short-range circulations induced by TB
[77]	Simultaneous pumping and mixing	Introducing two opposing microelectrode arrays placed at an angle relative to channel length	Mixing time reduced by 95% compared to diffusion-limited methods
[72]	Mixing	Study of light induced ACET flow over electrodes of different materials using opposing electrodes	Electrodes with high optical absorption rate and low thermal conductivity are best for effective light-induced heating
[58]	Comprehensive particle and droplet manipulation	Combining ACET and DEP	Particle transit time between multiple branches 0.008 s; droplet sorting purity 90%; particle sorting purity 93%
